# Author Correction: Generation of the organotypic kidney structure by integrating pluripotent stem cell-derived renal stroma

**DOI:** 10.1038/s41467-023-37484-y

**Published:** 2023-04-04

**Authors:** Shunsuke Tanigawa, Etsuko Tanaka, Koichiro Miike, Tomoko Ohmori, Daisuke Inoue, Chen-Leng Cai, Atsuhiro Taguchi, Akio Kobayashi, Ryuichi Nishinakamura

**Affiliations:** 1 Department of Kidney Development, Institute of Molecular Embryology and Genetics, Kumamoto University, Kumamoto, 860-0811 Japan; 2 Department of Pediatrics, Indiana University School of Medicine, Indianapolis, IN 46202 USA; 3 Present Address: Department of Genome Regulation, Max Planck Institute for Molecular Genetics, Berlin, Germany

**Keywords:** Stem-cell differentiation, Organogenesis, Kidney, Cell signalling

Correction to: *Nature Communications* 10.1038/s41467-022-28226-7, published online 01 February 2022

The original version of the Supplementary Information associated with this Article contained an error in the legend of Supplementary Fig. 1d, which stated ‘Tamoxifen was injected at E9.5 and the mice were analyzed at E15.5’. The correct version of the legend now states ‘Tamoxifen was injected at E11.5 and the mice were analyzed at E15.5’ The HTML has been updated to include a corrected version of the Supplementary Information.

## Supplementary information

Update Supplementary Information

