# Supplementary material for: Generation of the organotypic kidney structure by integrating pluripotent stem cell-derived renal stroma
Source: Nat Commun. 2022 Feb 1;13:611. doi: 10.1038/s41467-022-28226-7 (PMC8807595; doi:10.1038/s41467-022-28226-7)
Supplement: Supplementary file 1 — Supplementary Information [file 41467_2022_28226_MOESM1_ESM.pdf]

Generation of the organotypic kidney structure by integrating pluripotent stem cell-derived renal stroma

Shunsuke Tanigawa[1#], Etsuko Tanaka[1#], Koichiro Miike[1], Tomoko Ohmori[1], Daisuke Inoue[1], Chen-Leng Cai[2], Atsuhiro Taguchi[1,3], Akio Kobayashi[1], and Ryuichi Nishinakamura[1]*

[1]Department of Kidney Development, Institute of Molecular Embryology and Genetics, Kumamoto University, Kumamoto 860-0811, Japan

[2] Department of Pediatrics, Indiana University School of Medicine, Indianapolis, IN 46202, USA

[3]Present address: Department of Genome Regulation, Max Planck Institute for Molecular Genetics, Berlin, Germany

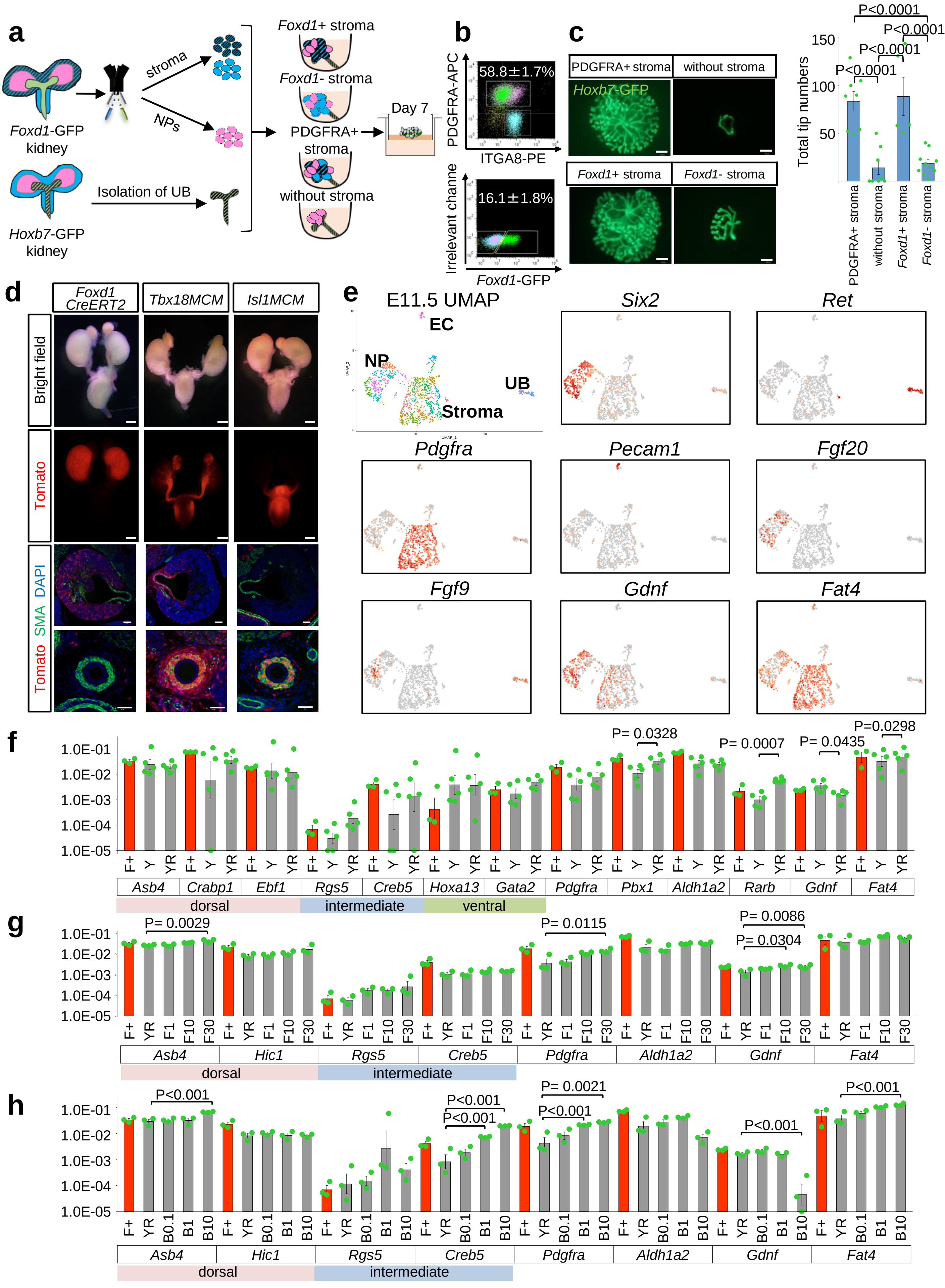
a
Foxd1-GFP kidney
Hoxb7-GFP kidney
stroma
NPs
Isolation of UB
Foxd1+ stroma
Foxd1- stroma
PDGFRA+ stroma
without stroma
Day 7
b
PDGFRA-APC
ITGA8-PE
58.8±1.7%
Irrelevant channe
Foxd1-GFP
16.1±1.8%
c
PDGFRA+ stroma
without stroma
Hoxb7-GFP
Foxd1+ stroma
Foxd1- stroma
Total tip numbers
150
100
50
P<0.0001
P<0.0001
P<0.0001
P<0.0001
PDGFRA+ stroma
without stroma
Foxd1+ stroma
Foxd1- stroma
d
Foxd1 CreERT2
Tbx18MCM
Isl1MCM
Bright field
Tomato
Tomato SMA DAPI
e
E11.5 UMAP
EC
NP
UB
Stroma
Six2
Ret
Pdgfra
Pecam1
Fgf20
Fgf9
Gdnf
Fat4
f
1.0E-01
1.0E-02
1.0E-03
1.0E-04
1.0E-05
P= 0.0328
P= 0.0007
P= 0.0435
P=0.0298
F+ Y YR
Asb4
Crabp1
Ebf1
Rgs5
Creb5
Hoxa13
Gata2
Pdgfra
Pbx1
Aldh1a2
Rarb
Gdnf
Fat4
dorsal
intermediate
ventral
g
P= 0.0029
P= 0.0115
P= 0.0086
P= 0.0304
F+ YR F1 F10 F30
Asb4
Hic1
Rgs5
Creb5
Pdgfra
Aldh1a2
Gdnf
Fat4
dorsal
intermediate
h
P<0.001
P<0.001
P<0.001
P= 0.0021
P<0.001
P<0.001
P<0.001
F+ YR B0.1 B1 B10
Asb4
Hic1
Rgs5
Creb5
Pdgfra
Aldh1a2
Gdnf
Fat4
dorsal
intermediate

Supplementary Figure 1

RA, FGF, and BMP signaling regulates dorsoventral patterning of renal SPs

(a) Schematic diagram of the aggregation assay for UB branching. The indicated stromal cell fraction and NPs isolated from *Foxd1*-GFP mouse kidneys (E11.5) were combined with UBs from *Hoxb7-GFP* kidneys (E11.5). The re-aggregates were cultured at the air/liquid interface for 7 days.

(b) Flow cytometric analysis of the *Foxd1-GFP* kidney at E11.5 before re-aggregation.

(c) UB branching of the re-aggregates with the indicated stromal cells or without stroma. The bar graph on the right shows the UB tip numbers in individual organoids (n=8, 8, 5, and 10 biologically independent samples, respectively). The Tukey–Kramer test (two-sided) was performed. Data are shown as mean ± SEM. Source data are provided as a Source Data file. Scale bars: 200 μm.

(d) Lineage-tracing analysis for each domain in the stroma using *Foxd1CreERT2*, *Tbx18MerCreMer* (*Tbx18MCM*), and *Isl1MerCreMer* (*Isl1MCM*) mice. Tamoxifen was injected at E11.5 and the mice were analyzed at E15.5. 1st row: bright-field photographs under stereomicroscopy; 2nd row: tdTomato fluorescence under stereomicroscopy; 3rd and 4th rows: kidney and ureter sections stained for tdTomato and smooth muscle actin (SMA). Scale bars: 1st and 2nd rows, 500 μm; 3rd row, 100 μm; 4th row, 50 μm. For each genotype, two biologically independent mice were examined in two separate experiments.

(e) UMAP plots of the E11.5 mouse kidney. *Fgf20* is expressed in NPs, while *Fgf9* is expressed in NPs and Ubs. *Gdnf* and *Fat4* are expressed in NPs and SPs.

(f–h) Expression of stromal domain-related genes after the culture, analyzed by qRT-PCR. (f) RA induces *Pbx1*, *Rarb*, and *Fat4*, in addition to dorsal SP-related genes (n=5 biologically independent experiments). (g, h) RA and FGF9 induce dorsal SP-related genes (including *Asb4*) and *Gdnf* (g), while RA and BMP4 induce intermediate and ventral SP-related genes (including *Creb5*) but reduce *Gdnf* (n=3) (h). Data are shown as mean ± SEM. Two-sided Student’s *t*-test was performed in (f) and Dunnett’s multiple comparison test (two-sided) was performed in (g, h). Source data are provided as a Source Data file. F+: Foxd1-GFP^{+}/PDGFRA^{+} dorsal SPs harvested at E11.5 (non-cultured; presented as a reference); Y: Y27632 (10 μM); R: RA (0.1 μM); F1: FGF9 (1 ng/ml) with YR; F10: FGF9 (10 ng/ml) with YR; F30: FGF9 (30 ng/ml) with YR; B0.1: BMP4 (0.1 ng/ml) with YR; B1: BMP4 (1 ng/ml) with YR; B10: BMP4 (10 ng/ml) with YR.

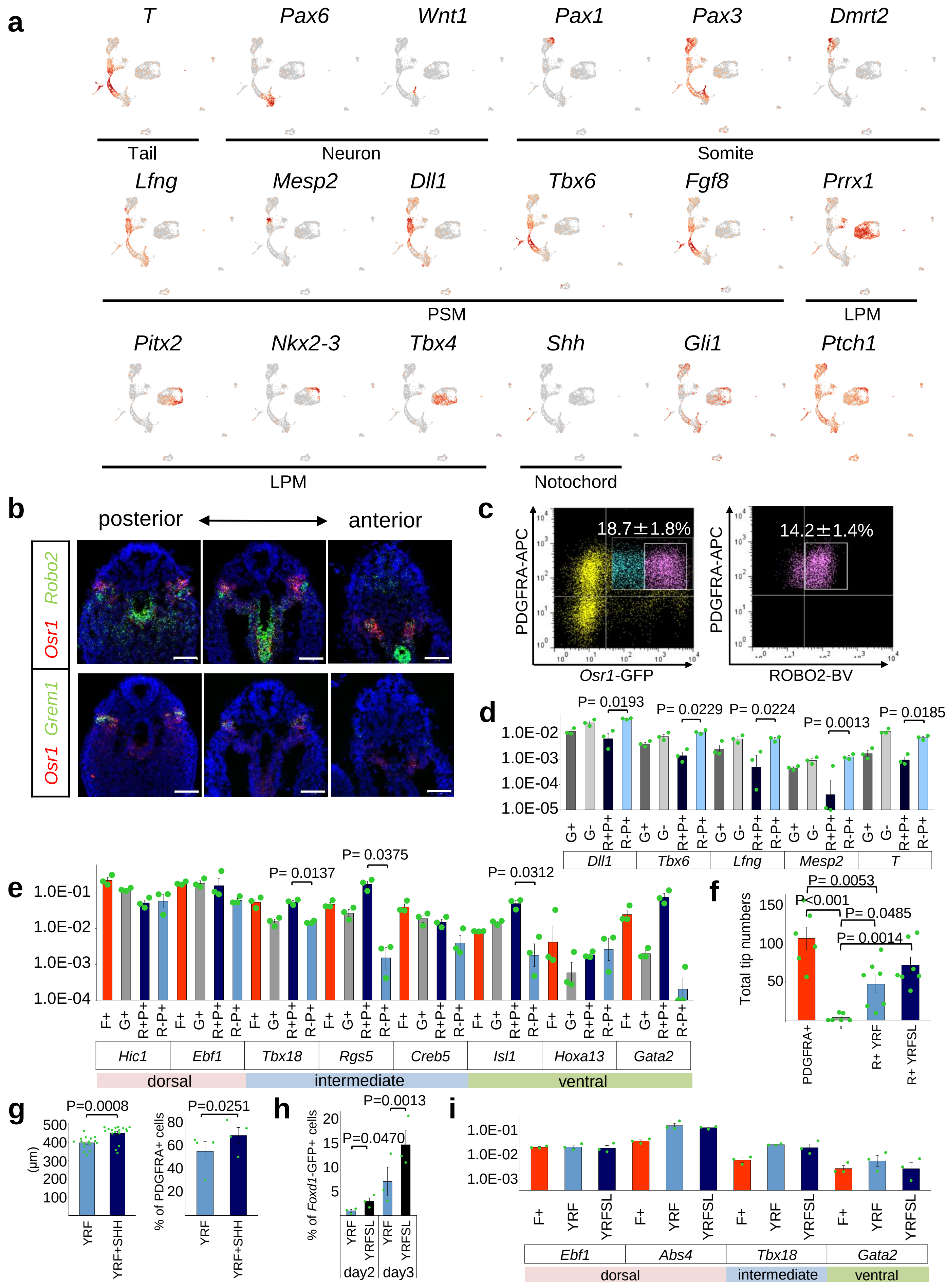

a
T
Pax6
Wnt1
Pax1
Pax3
Dmrt2
Tail
Neuron
Somite
Lfng
Mesp2
Dll1
Tbx6
Fgf8
Prrx1
PSM
LPM
Pitx2
Nkx2-3
Tbx4
Shh
Gli1
Ptch1
LPM
Notochord
b
posterior
anterior
Osr1 Robo2
Osr1 Grem1
c
18.7±1.8%
14.2±1.4%
PDGFRA-APC
Osr1-GFP
ROBO2-BV
d
P= 0.0193
P= 0.0229
P= 0.0224
P= 0.0013
P= 0.0185
G+ G- R+P+ R-P+
Dll1
Tbx6
Lfng
Mesp2
T
e
P= 0.0137
P= 0.0375
P= 0.0312
F+ G+ R+P+ R-P+
Hic1
Ebf1
Tbx18
Rgs5
Creb5
Isl1
Hoxa13
Gata2
dorsal
intermediate
ventral
f
P= 0.0053
P<0.001
P= 0.0485
P= 0.0014
Total tip numbers
PDGFRA+
-
R+ YRF
R+ YRFSL
g
P=0.0008
P=0.0251
(μm)
% of PDGFRA+ cells
YRF
YRF+SHH
h
P=0.0013
P=0.0470
% of Foxd1-GFP+ cells
YRF
YRFSL
day2
day3
i
F+
YRF
YRFSL
Ebf1
Abs4
Tbx18
Gata2
dorsal
intermediate
ventral

Supplementary Figure 2

ROBO2$^+$PDGFRA$^+$ IM is induced to dorsal SPs *in vitro*

(a) UMAP plots of genes expressed in the posterior part (caudal from the 26th somite) of the E9.5 mouse embryo. Genes in the tailbud mesenchyme (*T*), neurons (*Pax6*, *Wnt1*), somites (*Pax1*, *Pax3*, *Dmrt2*), LPM (*Prrx1*, *Pitx2*, *Nkx2-3*, *Tbx4*), PSM (*Lfng*, *Mesp2*, *Dll1*, *Tbx6*, *Fgf8*), and notochord (*Shh*), as well as Shh downstream genes (*Gli1*, *Ptch1*), are shown.

(b) *In situ* hybridization of *Osr1*, *Robo2*, and *Grem1* in the E9.5 mouse embryo. The left columns represent the *Osr1*$^+$/*Robo2*$^+$/*Grem1*$^+$ posterior IM, while the right columns show the more anterior part. The dorsal side faces upward, while the ventral side faces downward. Scale bars: 100 μm. Two biologically independent mice were examined in two separate experiments.

(c) Flow cytometric analysis of *Osr1-GFP* embryos at E9.5. The Osr1-GFP^{high}PDGFRA$^+$ fraction is mostly ROBO2$^+$.

(d) Expression of PSM-related genes in the ROBO2−PDGFRA$^+$ and Osr1-GFP$^-$PDGFRA$^+$ fractions. G+: Osr1-GFP$^+$ cells; G−: Osr1-GFP- cells; R+P+: ROBO2$^+$PDGFRA$^+$ cells; R−P+: ROBO2-/PDGFRA$^+$ cells. Data are shown as mean ± SEM (n=3 biologically independent experiments). Two-sided Student's *t*-test was performed.

(e) Expression of stromal domain-related genes after a 2-day culture of the indicated cell fractions in the YRF condition. F+: Foxd1-GFP$^+$PDGFRA$^+$ dorsal SPs at E11.5 (non-cultured; presented as a reference). Data are shown as mean ± SEM (n=3 biologically independent experiments). Two-sided Student's *t*-test was performed.

(f) UB branch numbers of the aggregates in Figure 2i. Data are shown as mean ± SEM (n=6, 6, 7, and 7 biologically independent samples, respectively). The Tukey–Kramer test (two-sided) was performed.

(g) Sphere sizes and percentages of induced PDGFRA$^+$ cells with or without Shh treatment. Data are shown as mean ± SEM. For the left graph, 16 and 17 spheres were analyzed under the indicated conditions, respectively. Four independent experiments were performed for the right graph. Two-sided Student's *t*-test was performed.

(h) Induction frequencies of Foxd1-GFP$^+$ cells from the ROBO2$^+$PDGFRA$^+$ fraction in E9.5 Foxd1-GFP embryos. Note the higher induction rates in the YRFSL condition compared with the YRF condition. Data are shown as mean ± SEM (n=3). Two-sided Student's *t*-test was performed.

(i) Expression of genes related to dorsoventral patterning in GFP$^+$ cells cultured from the ROBO2$^+$PDGFRA$^+$ fraction in E9.5 Foxd1-GFP embryos. Appended to Figure 2j. Data are shown as mean ± SEM (n=3). Two-sided Student's *t*-test was performed, but no statistical differences were detected.

(d–i) The source data are provided as a Source Data file.

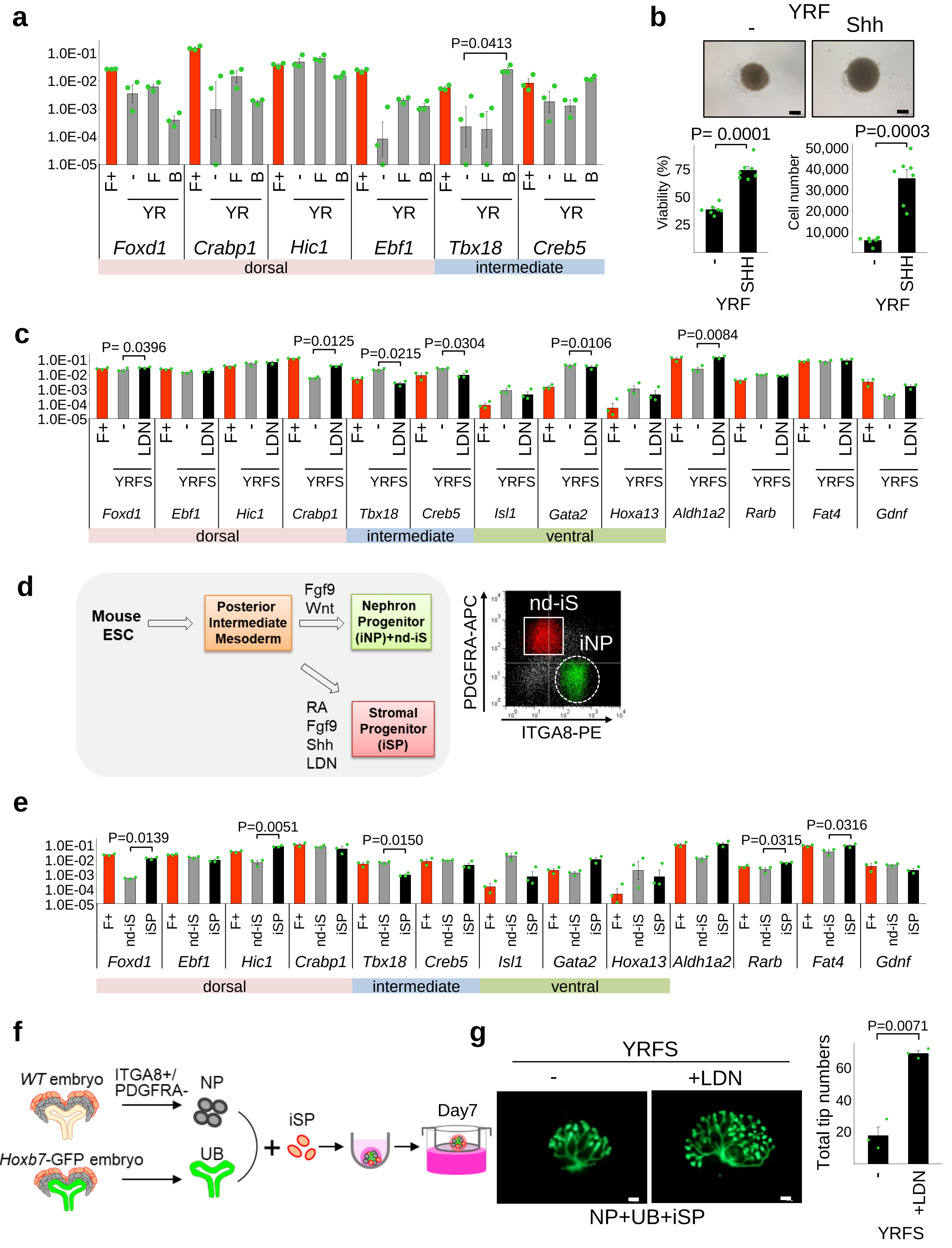

a
1.0E-01
1.0E-02
1.0E-03
1.0E-04
1.0E-05
P=0.0413
F+ - F B
YR
Foxd1
Crabp1
Hic1
Ebf1
Tbx18
Creb5
dorsal
intermediate
b
YRF
-
Shh
P= 0.0001
Viability (%)
75
50
25
-
SHH
YRF
P=0.0003
Cell number
50,000
40,000
30,000
20,000
10,000
-
SHH
YRF
c
P= 0.0396
P=0.0125
P=0.0215
P=0.0304
P=0.0106
P=0.0084
1.0E-01
1.0E-02
1.0E-03
1.0E-04
1.0E-05
F+ - LDN
YRFS
Foxd1
Ebf1
Hic1
Crabp1
Tbx18
Creb5
Isl1
Gata2
Hoxa13
Aldh1a2
Rarb
Fat4
Gdnf
dorsal
intermediate
ventral
d
Mouse ESC
Posterior Intermediate Mesoderm
Fgf9
Wnt
Nephron Progenitor (iNP)+nd-iS
RA
Fgf9
Shh
LDN
Stromal Progenitor (iSP)
PDGFRA-APC
nd-iS
iNP
ITGA8-PE
e
P=0.0139
P=0.0051
P=0.0150
P=0.0315
P=0.0316
1.0E-01
1.0E-02
1.0E-03
1.0E-04
1.0E-05
F+ nd-iS iSP
Foxd1
Ebf1
Hic1
Crabp1
Tbx18
Creb5
Isl1
Gata2
Hoxa13
Aldh1a2
Rarb
Fat4
Gdnf
dorsal
intermediate
ventral
f
WT embryo
ITGA8+/
PDGFRA-
NP
Hoxb7-GFP embryo
UB
iSP
Day7
g
YRFS
-
+LDN
NP+UB+iSP
P=0.0071
Total tip numbers
60
40
20
-
+LDN
YRFS

Supplementary Figure 3

Induction of dorsal SPs from mouse ESCs

(a) Effects of FGF9 and BMP4 on SP induction from mouse ESCs. Expression of SP-related genes after culture, analyzed by qRT-PCR. F+: Foxd1-GFP^{+}PDGFRA^{+} dorsal SPs harvested at E11.5 (non-cultured; presented as a reference); Y: Y27632 (10 μM); R: RA (0.1 μM); F: FGF9 (10 ng/ml); B: BMP4 (10 ng/ml). Data are shown as mean ± SEM (n=3 biologically independent experiments). Two-sided Student's *t*-test was performed.

(b) Effects of SHH addition to the YRF condition. The right graph shows the numbers of PDGFRA+ cells in the spheres. The lower graphs show the viability and cell numbers of the spheres. Data are shown as mean ± SEM (n=7 biologically independent samples). Two-sided Student's *t*-test was performed. Scale bars: 100 μm.

(c) Effects of LDN193189 addition on expression of stromal domain-related genes at day 9.5, analyzed by qRT-PCR. F+: Foxd1-GFP^{+}PDGFRA^{+} cells harvested from E11.5 kidneys (non-cultured; presented as a reference). Data are shown as mean ± SEM (n=3 biologically independent experiments). Two-sided Student's *t*-test was performed.

(d) Schematic diagram of NP induction from mouse ESCs. Right panel: flow cytometric analysis of mouse ESC-derived nd-iS, co-induced with NPs.

(e) Comparison of SP-related gene expression between nd-iS and iSPs derived from another mouse ESC line (G4-2). Data are shown as mean ± SEM (n=3 biologically independent experiments). Two-sided Student's *t*-test was performed.

(f) Schematic diagram of the aggregation assay. Mouse ESC-derived iSPs are combined with E11.5 embryo-derived ITGA8^{+}PDGFRA^{-} NPs and Hoxb7-GFP^{+} UBs, followed by a 7-day culture at the air/liquid interface.

(g) Enhanced UB branching in the aggregates with iSPs induced in the presence of LDN193189. Data are shown as mean ± SEM (n=3 biologically independent experiments). Two-sided Student's *t*-test was performed. (a-c, e, g) Source data are provided as a Source Data file.

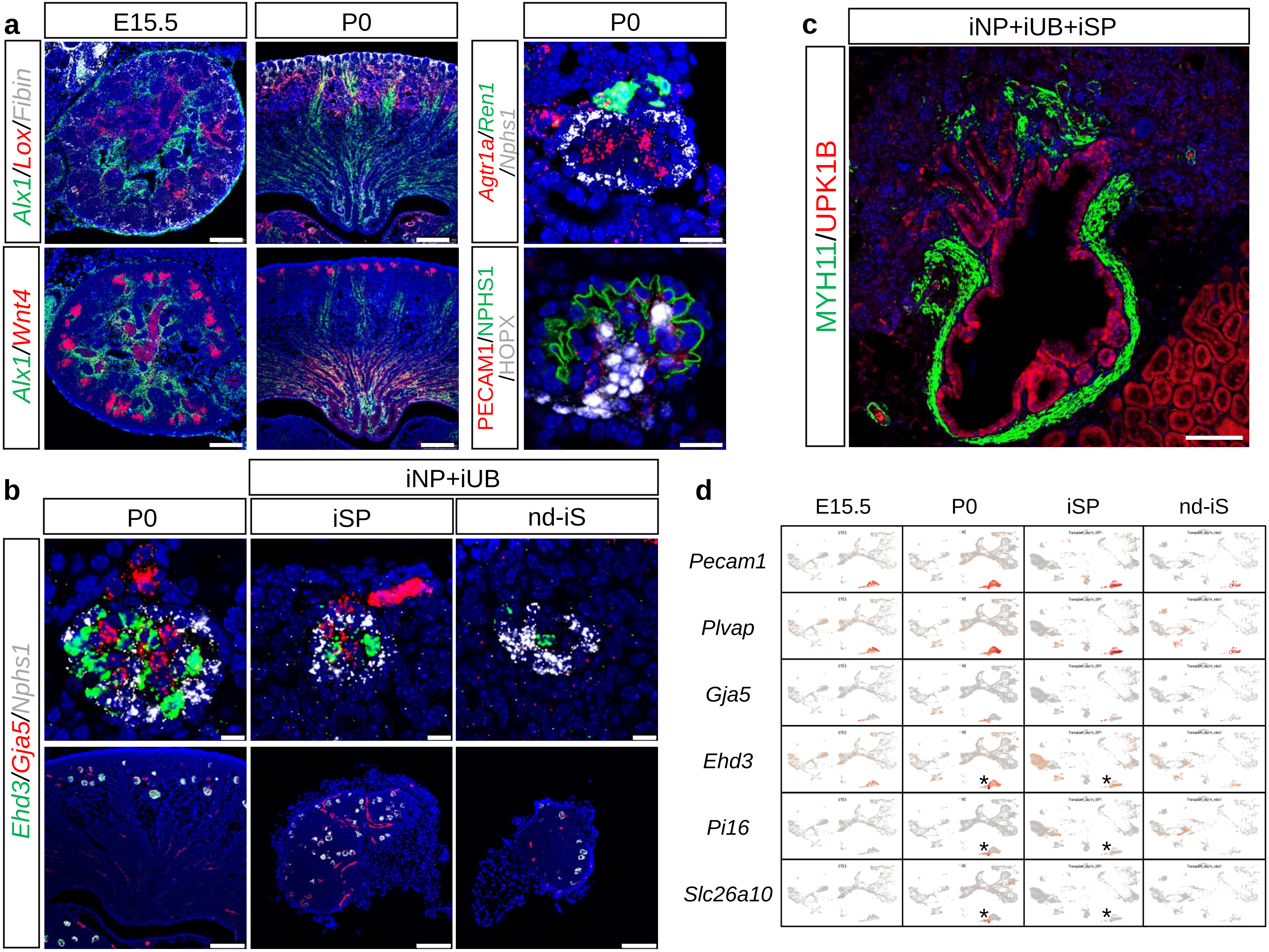

a
E15.5
P0
P0
Alx1/Lox/Fibin
Agtr1a/Ren1/Nphs1
Alx1/Wnt4
PECAM1/NPHS1/HOPX
c
iNP+iUB+iSP
MYH11/UPK1B
b
iNP+iUB
P0
iSP
nd-iS
Ehd3/Gja5/Nphs1
d
E15.5
P0
iSP
nd-iS
Pecam1
Plvap
Gja5
Ehd3
Pi16
Slc26a10

Supplementary Figure 4

Analysis of embryonic kidneys and mouse ESC-derived transplanted organoids

(a) Expression of stromal markers in embryonic kidneys. Left panels: *in situ* hybridization of E15.5 and P0 kidneys. Scale bars: 250 μm. Right upper panel: *in situ* hybridization of glomerulus at P0. Right lower panel: immunostaining of glomerulus at P0. Scale bar: 25 μm. Two biologically independent mice were examined in two separate experiments.

(b) *In situ* hybridization of endothelial marker genes in neonatal (P0) kidneys and transplanted organoids (14 days post-transplantation). Scale bar: 25 μm. Six organoids in each condition obtained from three independent transplantation experiments were analyzed. For the P0 kidneys, two biologically independent mice were examined in two separate experiments.

(c) A dilated ureter in the iSP-derived organoids at day 28 post-transplantation. Scale bar: 100 μm. Two organoids obtained from two independent transplantation experiments were analyzed.

(d) UMAP plots of representative genes for ECs in the embryonic kidneys (E15.5 and P0) and transplanted organoids (generated using iSPs or nd-iS). *: glomerular EC clusters.

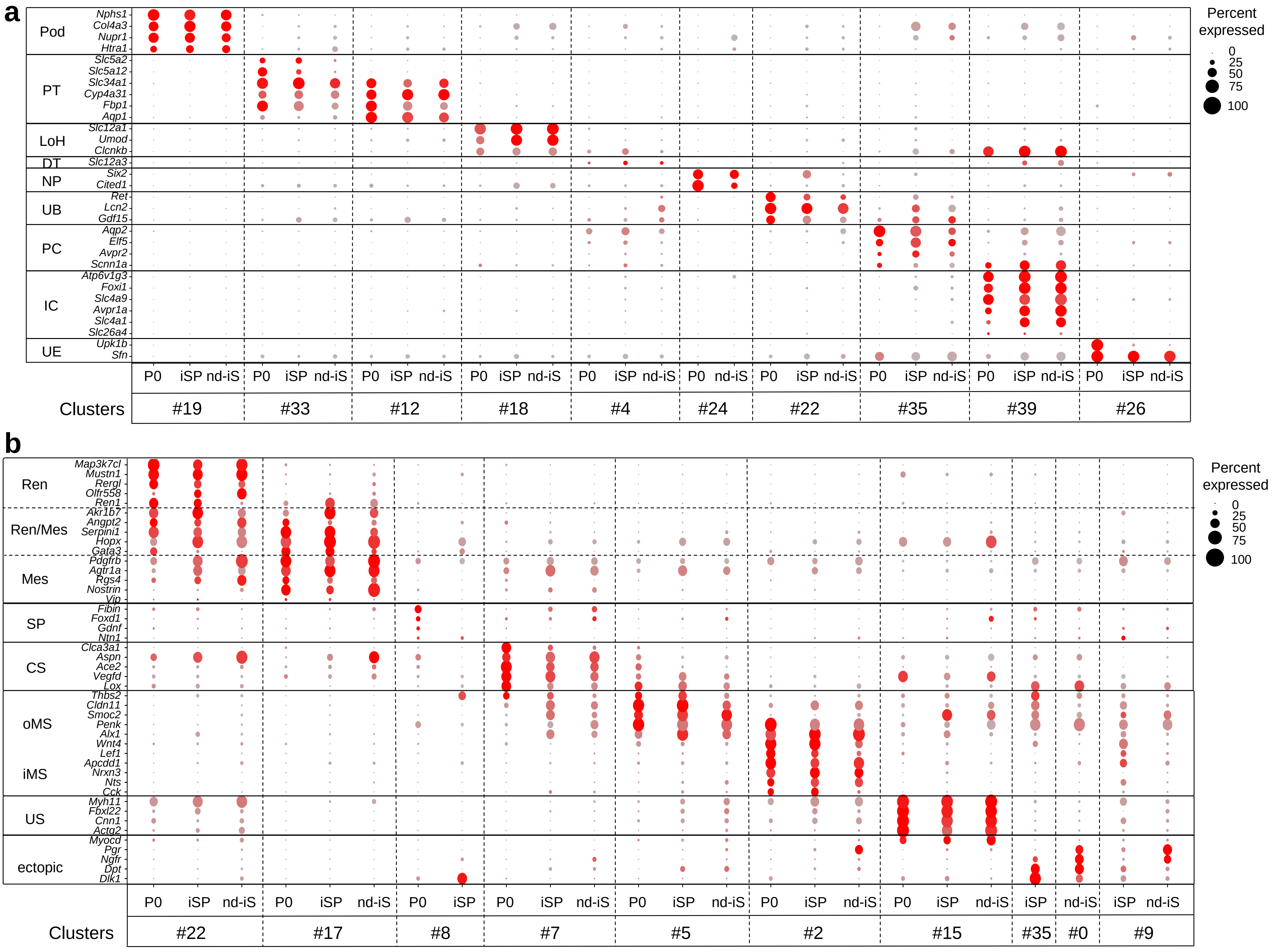

a
Pod
Nphs1
Col4a3
Nupr1
Htra1
PT
Slc5a2
Slc5a12
Slc34a1
Cyp4a31
Fbp1
Aqp1
LoH
Slc12a1
Umod
Clcnkb
DT
Slc12a3
NP
Six2
Cited1
UB
Ret
Lcn2
Gdf15
PC
Aqp2
Elf5
Avpr2
Scnn1a
IC
Atp6v1g3
Foxi1
Slc4a9
Avpr1a
Slc4a1
Slc26a4
UE
Upk1b
Sfn
Percent expressed
0
25
50
75
100
P0 iSP nd-iS
Clusters
#19 #33 #12 #18 #4 #24 #22 #35 #39 #26
b
Ren
Map3k7cl
Mustn1
Rergl
Olfr558
Ren1
Ren/Mes
Akr1b7
Angpt2
Serpini1
Hopx
Gata3
Mes
Pdgfrb
Agtr1a
Rgs4
Nostrin
Vip
SP
Fibin
Foxd1
Gdnf
Ntn1
CS
Clca3a1
Aspn
Ace2
Vegfd
Lox
oMS
Thbs2
Cldn11
Smoc2
Penk
Alx1
Wnt4
iMS
Lef1
Apcdd1
Nrxn3
Nts
Cck
US
Myh11
Fbxl22
Cnn1
Actg2
ectopic
Myocd
Pgr
Ngfr
Dpt
Dlk1
Percent expressed
0
25
50
75
100
P0 iSP nd-iS
Clusters
#22 #17 #8 #7 #5 #2 #15 #35 #0 #9

Supplementary Figure 5

Nephron, UB, and stromal lineages in the ESC-derived kidney mature close to the neonatal stage upon transplantation

(a) Dot plots of representative genes in the nephron and UB clusters in the P0 kidney, iSP-derived organoids, and nd-iS-derived organoids. Cluster 24 (#24) is absent in the iSP-derived organoids. Pod: podocyte; PT: proximal tubule; LoH: loop of Henle; DT: distal tubule; PC: principal cell; IC: intercalated cell; UE: uroepithelium.

(b) Dot plots of representative genes in the stromal clusters in the P0 kidney, iSP-derived organoids, and nd-iS-derived organoids. Organoid-specific genes are also shown. Cluster 8 (#8) is absent in the nd-iS-derived organoids. Clusters 35, 0, and 9 are only present in the organoids. Ren: renin cell; Mes: mesangial cell; CS: cortical stroma; oMS: outer medullary stroma; iMS: inner medullary stroma; US: ureteric stroma.

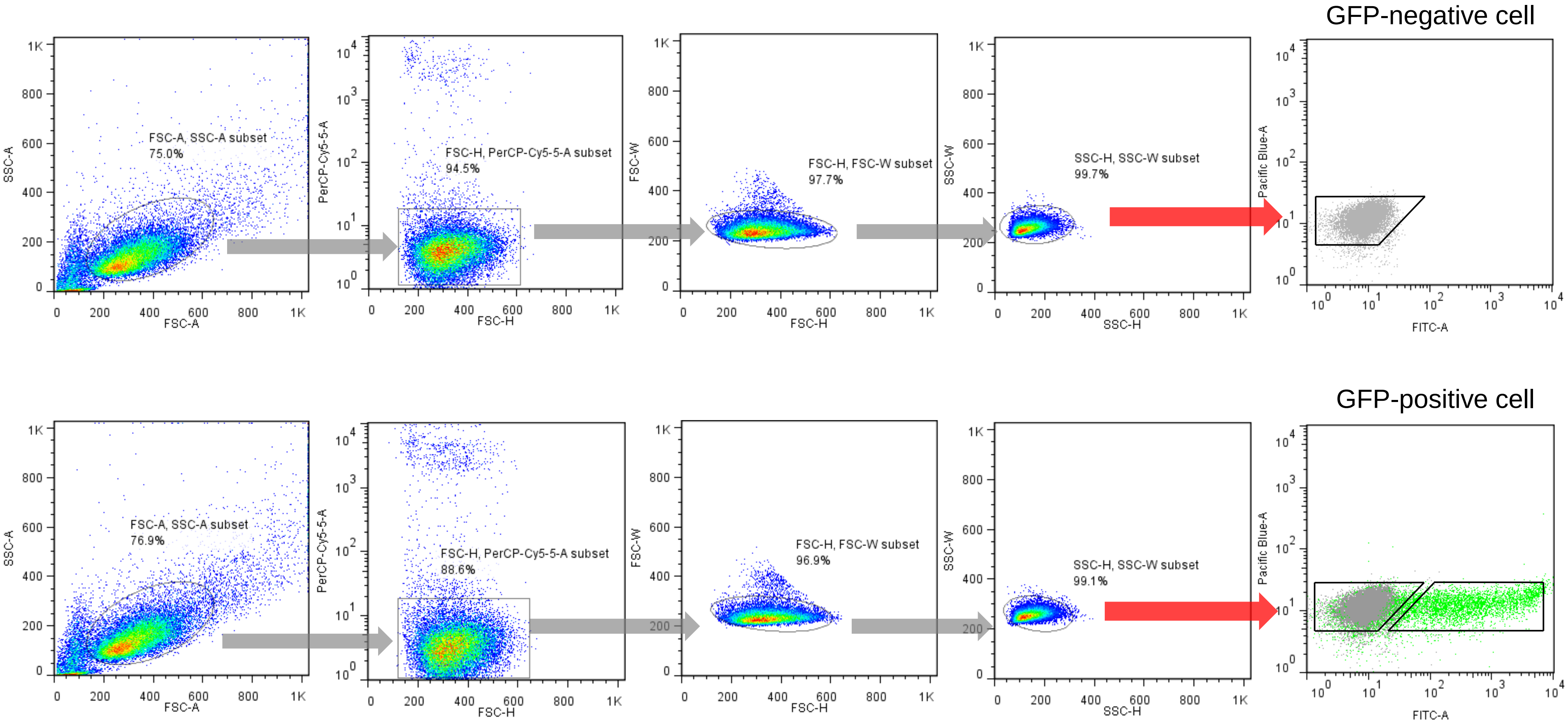
GFP-negative cell
FSC-A, SSC-A subset
75.0%
FSC-H, PerCP-Cy5-5-A subset
94.5%
FSC-H, FSC-W subset
97.7%
SSC-H, SSC-W subset
99.7%
GFP-positive cell
FSC-A, SSC-A subset
76.9%
FSC-H, PerCP-Cy5-5-A subset
88.6%
FSC-H, FSC-W subset
96.9%
SSC-H, SSC-W subset
99.1%
SSC-A
FSC-A
PerCP-Cy5-5-A
FSC-H
FSC-W
SSC-W
SSC-H
Pacific Blue-A
FITC-A

Supplementary Figure 6

FACS sequential gating/sorting strategies

Live cells were initially gated with the FSC/SSC and PI-negative population, and then FSC-H/FSC-W was applied to exclude doublet cells. The isolated cell populations were subsequently gated with cell surface markers or GFP^+ cells as indicated in the Figures.

Supplementary Table 1: Reagent list

REAGENT or RESOURCE	SOURCE	IDENTIFIER	Dilution
Primary Antibodies			
KRT8 (TROMA-I)	DSHB	N/A	1:50
SIX2	Proteintech	Cat.# 11562-1-AP	1:100
FOXD1	Santa Cruz	Cat.# sc-47585	1:500
TBX18	Santa Cruz	Cat.# sc-17869	1:500
ISL1	Abcam	Cat.# ab20670	1:200
SMA	Dako	Cat.# M0851	1:100
RFP	Rockland	Cat.# 600-401-379	1:100
LTL	VECTOR	Cat.# B-1325	1:200
CDH1	BD	Cat.# 610181	1:100
NPHS1	Progen	Cat.# GP-N2	1:100
CAR2	Santa Cruz	Cat.# sc-25596	1:100
AQP2	Sigma	Cat.# A7310	1:100
SLC12A1	StressMarq Bioscience	Cat.# SPC-401D	1:100
HOPX	Proteintech	Cat.# 11419-1-AP	1:500
Mouse PECAM1	Dianova	Cat.# DIA-310	1:50
MYH11	Abcam	Cat.# Ab53219	1:100
UPK1B	Sigma	Cat.# WH0007348M2	1:200
ITGA8	R&D	Cat.# BAF4076	1:50
PDGFRA	Biolegend	Cat.# 135907	1:500
ROBO2	R&D	Cat.# BAF3147	1:200
Fluorescence-conjugated secondary antibodies			
BV421 Streptoavidin	Biolegend	Cat.# 405226	1:100
PE Streptoavidin	BD Pharmingen	Cat.# 554061	1:100
Alexa 488 donkey anti-rabbit IgG	Thermo	A21206	1:500
Alexa 488 chicken anti-mouse IgG	Thermo	A21200	1:500
Alexa 488 donkey anti-goat IgG	Thermo	A11055	1:500
Alexa 488 chicken anti-rat IgG	Thermo	A21470	1:500
Alexa 568 donkey anti-rabbit IgG	Thermo	A11042	1:500
Alexa 568 donkey anti-mouse IgG	Thermo	A11037	1:500
Alexa 568 donkey anti-goat IgG	Thermo	A11057	1:500
Alexa 568 goat anti-guinea pig IgG	Thermo	A11075	1:500

Alexa 568 Streptoavidin	Thermo	A21094	1:500
Alexa 633 goat anti-rat IgG	Thermo	A21094	1:500
Alexa 633 goat anti-guinea pig IgG	Thermo	A21105	1:500
Alexa 633 goat anti-mouse IgG	Thermo	A21052	1:500
Alexa 633 Streptoavidin	Thermo	S11226	1:500
Chemicals, Peptides, and Recombinant Proteins			
Y27632	Wako	Cat.# 257-00511	
human FGF2	Wako	Cat.# 064-04541	
LDN193189	Wako	Cat.# 126-05851	
CHIR99021	Axon	Cat.# AXN1386-00	
PD0325901	Wako	Cat.# 162-25291	
Retinoic acid	Sigma	Cat.# R-2625-50MG	
Activin A	R&D	Cat.# 338-AC	
human BMP4	R&D	Cat.# 314-BP	
human FGF9	R&D	Cat.# 273-F9	
human FGF acidic	R&D	Cat.# aa 16-155	
human FGF basic	R&D	Cat.# 233-FB-025	
human GDNF	R&D	Cat.# 212-GD-010	
Mouse SHH-N	R&D	Cat.# 461-SH-025	
TTNPB	SIGMA	T3757	
JAK inhibitor I	MIllipore	420099	
A83-01	TOCRIS	2939	
SB202190	SIGMA	S7067	
Critical Commercial Assays			
RNeasy Plus Micro Kit	QIAGEN	Cat.# 74034	
SuperScript VILO cDNA Synthesis Kit	ThermoFisher	Cat.# 11754250	
TB Green Fast qPCR Mix	Takara	Cat.# RR430A	
Experimental Models: Cell Lines			
Mouse ESC: Hoxb7-GFP line	Taguchi et al., 2017[1]	N/A	
Mouse ESC: G4-2 line	Niwa et al. 2000[2]	N/A	
Experimental Models: Organisms/Strains			

Mouse: Osr1-GFP	Taguchi et al, 2014[3]	N/A
Mouse: Hoxb7-GFP	Jackson Laboratory	Cat.# 016251
Mouse: Foxd1-GFPCre	Jackson Laboratory	Cat.# 012463
Mouse: Foxd1-GFPCreERT2	Jackson Laboratory	Cat.# 012464
Mouse: Isl1-MerCreMer	Jackson Laboratory	Cat.# 029566
Mouse: ROSA26-CAG-tdTomato	Jackson Laboratory	Cat.# 007905
Mouse: Tbx18-MerCreMer	Grisanti et al, 2013[4]	N/A
Mouse: NOD/ShiJic-scidJcl	CHARLES RIVER LABORATORIES JAPAN, INC.	N/A
Oligonucleotides		
See Table S4 for PCR primer sequences		
RNAscope Probe- Mm-Osr1	Advanced Cell Diagnostics	496281-C2
RNAscope Probe- Mm-Robo2	Advanced Cell Diagnostics	475961
RNAscope Probe- Mm-Grem1	Advanced Cell Diagnostics	314741-C3
RNAscope Probe- Mm-Wnt7b	Advanced Cell Diagnostics	401131
RNAscope Probe- Mm-Ret	Advanced Cell Diagnostics	431791-C2
RNAscope Probe- Mm-Six2	Advanced Cell Diagnostics	500011, 500011-C3
RNAscope Probe- Mm-Foxd1	Advanced Cell Diagnostics	495501-C3
RNAscope Probe- Mm-Alx1	Advanced Cell Diagnostics	403161, 403161-C3

RNAscope Probe- Mm-Lox	Advanced Cell Diagnostics	425311
RNAscope Probe- Mm-Fibin	Advanced Cell Diagnostics	545551-C2
RNAscope Probe- Mm-Wnt4	Advanced Cell Diagnostics	401101-C2
RNAscope Probe- Mm-Aqp2	Advanced Cell Diagnostics	452411-C3
RNAscope Probe- Mm-Ren1	Advanced Cell Diagnostics	433461-C2
RNAscope Probe- Mm-Agtr1a	Advanced Cell Diagnostics	481161-C3
RNAscope Probe- Mm-Nphs1	Advanced Cell Diagnostics	433571
RNAscope Probe- Mm-Ehd3	Advanced Cell Diagnostics	1110201
RNAscope Probe- Mm-Gja5	Advanced Cell Diagnostics	518041-C2
Software and Algorithms		
FlowJo (ver 7.6.5)	Tree Star	https://www.flowjo.com
Imaris (ver 7.7.0)	BITPLANE	http://www.bitplane.com/

Supplementary Table 2: Primer list for qRT-PCR

Oligonucleotides		
**mouse qRT-PCR Primers**	**Sequence Forward**	**Sequence Reverse**
Actb	CATCCGTAAAGACCTCTATGCCAAC	ATGGAGCCACCGATCCACA
Foxd1	TCGCTCTGTCTTGGCACTAGGA	ACGCCTGGACCTGAGAATCTCTAC
Crabp1	AGCAGCGAGAATTCGACGAG	CGCACAGTAGTGGATGTCTTGATG
Ebf1	GACTACGGCTTCCAGAGGTTACAGA	CCCATACAGGGCTTCAACCAG
Asb4	CACATTCGATGGAACATAAAGTGGA	CGAATGGCACATC1TGACAGG
Hic1	GCCCGGGACTGATAATGTGAAG	ACCTCGGAAGCAGAGACAGATG
Tbx18	ATCCCTGGAATCCCAAAGCAA	CCAGACAACAGGTGAGGATGTGTAG
Rgs5	AGTTCTGGGTTGCCTGTGAGAATTA	CCAAGTCAAAGCTGCGAGGA
Creb5	GTCCGATAACAGCCATGCAGAA	TCGCTGACCGATGAGGATGTAG
Isl1	TCCATGGCAAAGCAGTGGAG	CAGCACGGCTGAGGTAAATGATAA
Hoxa13	CCACCTCTGGAAGTCCACTCTG	CCGTTTGTCCTTGGTAATGAATTTG
Gata2	GGCTCTACCACAAGATGAATGGA	CGCCATAAGGTGGTGGT「GTC
Pdgfra	AGCAAACATCTTGACTTGGGAACA	ACTTGCATCATTCCCGGACAC
Pbx1	GCCCATGGAAGCCAAGCTAA	GTGACTGCACGCTCATGAACAA
Aldh1a2	AGCCCATTGGAGTGTGTGGAC	TGCTCAGCGGGTTTGATGAC
Rarb	AGCAAGTTAGTCTGCCGTCTGGA	AGGCCACTTGCCCATACCTTC
Gdnf	CACGTTTCGCATGGTTCCA	TGGGCAGCTGAGGTTGTCA
Fat4	GTGTTTAACGTCACCGATGCAGA	TCCACTGCAAACTGCCCAAG
Osr1	ACTGATGAGCGACCTTACACCTG	ACTTGTGAGTGTAGCGTCTTGTGGA
Wt1	TGAAGACCCACACCAGGACTC	TGTGATGGCGGACCAATTC
Six2	GCAACTTCCGCGAGCTCTAC	GCCTTGAGCCACAACTGCTG
Grem1	TTCAGTGCTCGCCCTATGCTC	TACACCCGCGGTCAAGTGAA
Pax2	AGGCATCAGAGCACATCAAATCAG	GGGTTGGCCGATGCAGATAG
Lfng	TTCATCGCCGTCAAGACCAC	CTTGGCCAGAGCTTCGTCCT
Dll1	CTGTCTGCCAGGGTGTGATGA	GCCATGGAGACAACCTGGGTA
Tbx6	GGTCAGCCTGAGCTTGGAGA	GGTCCAGGCCAGTGACTGATAC
Mesp2	ACTGGACACAATCCACTGAACCTG	TGAGGCTGTAGTCTCTGGCATGA
T	CCATGCTGCAGTCCCATGA	GCTCACAGACCAGAGACTGGGATAC
Tbx4	ACTGGACCGATGGATGACGAG	TGGCCACCCACAGAGATTAACA
Foxf1	GAGCAGCCATACCTTCACCAA	ACATGCTGGGCGACTGTGAG
Meox1	GATTGCATGGTACTTGGGACGA	TCCTTGGTGAAGGCTGTCCTC

1. Taguchi, A. & Nishinakamura, R. Higher-order kidney organogenesis from pluripotent stem cells. *Cell Stem Cell* **21**, 730–746 (2017).
2. Niwa, H., Miyazaki, J. I. & Smith, A. G. Quantitative expression of Oct-3/4 defines differentiation, dedifferentiation or self-renewal of ES cells. *Nat. Genet.* **24**, 372–376 (2000).
3. Taguchi, A. *et al.* Redefining the in vivo origin of metanephric nephron progenitors enables generation of complex kidney structures from pluripotent stem cells. *Cell Stem Cell* **14**, 53–67 (2014).
4. Grisanti, L. *et al.* Tbx18 targets dermal condensates for labeling, isolation, and gene ablation during embryonic hair follicle formation. *J. Invest. Dermatol.* **133**, 344–353 (2013).
