# Supplementary material for: Generation of the organotypic kidney structure by integrating pluripotent stem cell-derived renal stroma
Source: Nat Commun. 2022 Feb 1;13:611. doi: 10.1038/s41467-022-28226-7 (PMC8807595; doi:10.1038/s41467-022-28226-7)
Supplement: Supplementary file 3 — Description of Additional Supplementary Files [file 41467_2022_28226_MOESM3_ESM.docx]

**Description of Additional Supplementary Files**

Title: Supplementary Data 1.

Description: Representative gene expression in the stromal clusters of transplanted mouse organoids and embryonic kidneys (related to Supplementary Fig. S5)

Title: Supplementary Data 2.

Description: Unbiased hierarchal clustering analysis of the induced stroma and embryonic stroma (related to Fig. 6d)
