# Supplementary material for: Generation of the organotypic kidney structure by integrating pluripotent stem cell-derived renal stroma
Source: Nat Commun. 2022 Feb 1;13:611. doi: 10.1038/s41467-022-28226-7 (PMC8807595; doi:10.1038/s41467-022-28226-7)
Supplement: Supplementary file 6 — Reporting Summary [file 41467_2022_28226_MOESM6_ESM.pdf]

nature portfolio

Corresponding author(s): Ryuichi Nishinakamura

Last updated by author(s): Dec 21, 2021

Reporting Summary

Nature Portfolio wishes to improve the reproducibility of the work that we publish. This form provides structure for consistency and transparency in reporting. For further information on Nature Portfolio policies, see our Editorial Policies and the Editorial Policy Checklist.

Statistics

For all statistical analyses, confirm that the following items are present in the figure legend, table legend, main text, or Methods section.

n/a	Confirmed	
☐	☒	The exact sample size (n) for each experimental group/condition, given as a discrete number and unit of measurement
☐	☒	A statement on whether measurements were taken from distinct samples or whether the same sample was measured repeatedly
☐	☒	The statistical test(s) used AND whether they are one- or two-sided<br>*Only common tests should be described solely by name; describe more complex techniques in the Methods section.*
☒	☐	A description of all covariates tested
☒	☐	A description of any assumptions or corrections, such as tests of normality and adjustment for multiple comparisons
☐	☒	A full description of the statistical parameters including central tendency (e.g. means) or other basic estimates (e.g. regression coefficient) AND variation (e.g. standard deviation) or associated estimates of uncertainty (e.g. confidence intervals)
☐	☒	For null hypothesis testing, the test statistic (e.g. F, t, r) with confidence intervals, effect sizes, degrees of freedom and P value noted<br>*Give P values as exact values whenever suitable.*
☒	☐	For Bayesian analysis, information on the choice of priors and Markov chain Monte Carlo settings
☒	☐	For hierarchical and complex designs, identification of the appropriate level for tests and full reporting of outcomes
☒	☐	Estimates of effect sizes (e.g. Cohen's d, Pearson's r), indicating how they were calculated

Our web collection on statistics for biologists contains articles on many of the points above.

Software and code

Policy information about availability of computer code

Data collection	Sequence and data collection were done by using Chromium equipment (10x Genomics) .
Data analysis	-For single cell RNA sequence analyses, raw sequencing data for each sample was converted to matrices of expression counts by using the Cell Ranger software (10x Genomics; the version used for each sample are 2.0.0 for E9.5, 3.0.2 for E11.5, and 4.0.0 for integrated data).<br>-Statistical analyses were performed by using Seurat software (version 3.1.1 for E9.5 and E11.5, and version 4.0.0 for integrated data).<br>-The original FACS data sets were collected by using BD FACS Diva software (v8.0.1). Data analysis were performed with Flowjo software (ver 7.6.5, TreeStar).<br>-Three-dimensional fluorescence images were captured by confocal microscopy (TSC SP8; Leica) and reconstructed by Imaris (v 7.7.0, Bitplane).

For manuscripts utilizing custom algorithms or software that are central to the research but not yet described in published literature, software must be made available to editors and reviewers. We strongly encourage code deposition in a community repository (e.g. GitHub). See the Nature Portfolio guidelines for submitting code & software for further information.

Data

Policy information about availability of data

All manuscripts must include a data availability statement. This statement should provide the following information, where applicable:

- Accession codes, unique identifiers, or web links for publicly available datasets
- A description of any restrictions on data availability
- For clinical datasets or third party data, please ensure that the statement adheres to our policy

Figures 1, 2, 3, and 6 have associated scRNA-seq raw data, which have been deposited in the National Center for Biotechnology Information Gene Expression

Omnibus (GSE178263 [https://www.ncbi.nlm.nih.gov/geo/query/acc.cgi?acc=GSE178263]). The scRNA-seq data of E15.5 and P0 embryonic kidneys were described previously (GSE149134 [https://www.ncbi.nlm.nih.gov/geo/query/acc.cgi?acc=GSE149134])(Naganuma et al., 2021).

Field-specific reporting

Please select the one below that is the best fit for your research. If you are not sure, read the appropriate sections before making your selection.

- [x] Life sciences
- [ ] Behavioural & social sciences
- [ ] Ecological, evolutionary & environmental sciences

For a reference copy of the document with all sections, see nature.com/documents/nr-reporting-summary-flat.pdf

Life sciences study design

All studies must disclose on these points even when the disclosure is negative.

Sample size	All experiments in this study were performed with a sample size as large as possible and repeated at least three independent experiments to perform statistical analysis. Sample size was not pre-determined by applying statistical methods.
Data exclusions	No data were excluded from the analysis
Replication	All induction experiments in this study were independently repeated 3 times with replicates. The number of replicates of samples and types of statistical analysis were described in Figure legends and supplementary information.
Randomization	Samples were allocated into experimental groups based on induced conditions of the cultured cells.
Blinding	The investigators were not blinded during allocating different treatments to avoid cross contamination between samples.

Reporting for specific materials, systems and methods

We require information from authors about some types of materials, experimental systems and methods used in many studies. Here, indicate whether each material, system or method listed is relevant to your study. If you are not sure if a list item applies to your research, read the appropriate section before selecting a response.

Materials & experimental systems

n/a	Involved in the study	
[ ]	[x]	Antibodies
[ ]	[x]	Eukaryotic cell lines
[x]	[ ]	Palaeontology and archaeology
[ ]	[x]	Animals and other organisms
[x]	[ ]	Human research participants
[x]	[ ]	Clinical data
[x]	[ ]	Dual use research of concern

Methods

n/a	Involved in the study	
[x]	[ ]	ChIP-seq
[ ]	[x]	Flow cytometry
[x]	[ ]	MRI-based neuroimaging

Antibodies

Antibodies used	KRT8 (1:50, DSHB, TROMA-I); SIX2 (1:100, Proteintech, 11562-1-AP); FOXD1(1:500, Santa Cruz,sc-47585); TBX18(1:500, Santa Cruz,sc-17869); ISL1 (1:200, Abcam, ab20670) SMA (1:100, Dako, M0851); RFP (1:100, Rockland, 600-401-379), LTL (1:200, VECTOR, B-1325); CDH1(1:100, BD biosciences, 610181); NPHS1(1:100, Progen, GP-N2); CAR2 (1:100, Santa Cruz, sc-25596); AQP2(1:100, Sigma, A7310); SLC12A1 (1:100, StressMarq Bioscience, SPC-401D); HOPX (1:500, Proteintech, 11419-1-AP); Mouse PECAM1 (1:50, Dianova, DIA-310); MYH11 (1:100, Abcam, Ab53219); UPK1B (1:200, Sigma, WH0007348M2); ITGA8 (1:50, R&D systems, BAF4076); PDGFRA (1:500, Biolegend, 135907); ROBO2 (1:200, R&D systems ,BAF3147).<br>Secondary antibodies and dilutions used are as follows: PE Streptoavidin (1:100, BD biosciences, 554061) ; BV421 Streptoavidin (1:100, Biolegend, 405226); Alexa 488 donkey anti-rabbit IgG (Thermo, #A21206, 1:500); Alexa 488 chicken anti-mouse IgG (Thermo, #A21200, 1:500); Alexa 488 donkey anti-goat IgG (Thermo, #A11055, 1:500); Alexa 488 chicken anti-rat IgG (Thermo, #A21470, 1:500);Alexa 568 donkey anti-rabbit IgG (Thermo, #A11042, 1:500); Alexa 568 donkey anti-mouse IgG (Thermo, #A11037, 1:500); Alexa 568 donkey anti-goat IgG (Thermo, #A11057, 1:500); Alexa 568 goat anti-guinea pig IgG (Thermo, #A11075, 1:500); Alexa 568 Streptoavidin (Thermo, #A21094, 1:500); Alexa 633 goat anti-rat IgG (Thermo, #A21094, 1:500); Alexa 633 goat anti-guinea pig IgG (Thermo, #A21105, 1:500); Alexa 633 goat anti-mouse IgG (Thermo, #A21052, 1:500); Alexa 633 Streptoavidin (Thermo, #S11226, 1:500).<br>The Tyramide Signal Amplification (TSA) kit (Thermo) was used for detection of Foxd1, Tbx18 and Hopx.
Validation	1.SIX2 (1:100, Proteintech, 11562-1-AP);<br>The following antibodies were validated on manufacture's website:

https://www.ptglab.com/products/SIX2-Antibody-11562-1-AP.htm
We previously published use of this antibody in doi: 10.1016/j.stem.2017.10.011

2. FOXD1 (1:500, Santa Cruz,sc-47585);
The following antibodies were validated on website:
https://www.citeab.com/antibodies/794015-sc-47585-foxd1-antibody-c-19
Citations (9)

3. TBX18 (1:500, Santa Cruz,sc-17869);
The following antibodies were validated on manufacture's website:
https://www.scbt.com/p/tbx18-antibody-c-20?productCanUrl=tbx18-antibody-c-20&_requestid=3896205
Citations (4)

4. ISL1 (1:200, Abcam, ab20670)
The following antibodies were validated on manufacture's website:
https://www.abcam.com/islet-1-antibody-ab20670.html

5. KRT8 (1:50, DSHB, TROMA-I);
The following antibodies were validated on manufacture's website:
https://dshb.biology.uiowa.edu/TROMA-I
We previously published use of this antibody in doi: 10.1016/j.stem.2017.10.011

6. SMA (1:100, Dako, M0851);
The following antibodies were validated on website:
https://www.citeab.com/antibodies/2414737-m0851-actin-smooth-muscle-concentrate
Citations (1235)

7. RFP (1:100, Rockland, 600-401-379),
The following antibodies were validated on manufacture's website:
https://rockland-inc.com/store/Antibodies-to-GFP-and-Antibodies-to-RFP-600-401-379-O4L_24299.aspx
We previously published use of this antibody in doi: 10.1038/s41598-018-37793-z

8. LTL (1:200, VECTOR, B-1325);
The following antibodies were validated on manufacture's website:
https://vectorlabs.com/biotinylated-lotus-tetragonolobus-lectin-ltl.html
We previously published use of this antibody in doi: 10.1016/j.stem.2017.10.011

9. CDH1 (1:100, BD biosciences, 610181);
The following antibodies were validated on manufacture's website:
https://www.bdbiosciences.com/en-us/products/reagents/microscopy-imaging-reagents/immunofluorescence-reagents/purified-mouse-anti-e-cadherin.610181
We previously published use of this antibody in doi: 10.1016/j.stem.2017.10.011

10. NPHS1 (1:100, Progen, GP-N2);
The following antibodies were validated on manufacture's website:
https://www.progen.com/products/antibodies/anti-nephrin-guinea-pig-polyclonal-serum
We previously published use of this antibody in doi: 10.1016/j.stemcr.2018.08.003

11. CAR2 (1:100, Santa Cruz, sc-25596);
The following antibodies were validated on manufacture's website:
https://www.scbt.com/p/ca-ii-antibody-h-70
Citations (10)

12. AQP2(1:100, Sigma, A7310);
The following antibodies were validated on manufacture's website:
https://www.sigmaaldrich.com/JP/en/product/sigma/a7310
We previously published use of this antibody in doi: 10.1681/ASN.2020030378

13. SLC12A1 (1:100, StressMarq Bioscience, SPC-401D);
The following antibodies were validated on manufacture's website:
https://www.stressmarq.com/products/antibodies/polyclonal-antibodies/nkcc2-antibody-spc-401/?v=3e8d115eb4b3
We previously published use of this antibody in doi: 10.1016/j.stem.2017.10.011

14. HOPX (1:500, Proteintech, 11419-1-AP);
The following antibodies were validated on manufacture's website:
https://www.ptglab.com/products/HOPX-Antibody-11419-1-AP.htm

15. Mouse PECAM1 (1:50, Dianova, DIA-310);
The following antibodies were validated on manufacture's website:

https://www.dianova.com/en/shop/dia-310-anti-cd31-mssw-from-rat-sz31-unconj-for-mouse-ffpe-tissue/

16. MYH11 (1:100, Abcam, Ab53219);
The following antibodies were validated on manufacture's website:
https://www.abcam.com/smooth-muscle-myosin-heavy-chain-11-antibody-1g12-ab683.html

17. UPK1B (1:200, Sigma, WH0007348M2);
The following antibodies were validated on manufacture's website:
https://www.sigmaaldrich.com/JP/en/product/sigma/wh0007348m2

18. ITGA8 (1:50, R&D systems, BAF4076);
The following antibodies were validated on manufacture's website:
https://www.rndsystems.com/products/mouse-integrin-alpha8-biotinylated-antibody_baf4076
We previously published use of this antibody in doi: 10.1016/j.stem.2017.10.011

19. PE Streptoavidin (1:100, BD biosciences, 554061);
The following antibodies were validated on manufacture's website:
https://www.bdbiosciences.com/en-eu/products/reagents/flow-cytometry-reagents/research-reagents/single-color-antibodies-ruo/pe-streptavidin.554061
We previously published use of this antibody in doi: 10.1016/j.stem.2017.10.011

20. mouse PDGFRA (1:500, Biolegend, 135907);
The following antibodies were validated on manufacture's website:
https://www.biolegend.com/ja-jp/products/apc-anti-mouse-cd140a-antibody-6439?GroupID=BLG8105
We previously published use of this antibody in doi: 10.1016/j.stem.2017.10.011

21. ROBO2 (1:200, R&D systems ,BAF3147);
The following antibodies were validated on manufacture's website:
https://www.rndsystems.com/products/human-robo2-biotinylated-antibody_baf3147
We previously published use of this antibody in doi: 10.1681/ASN.2018070747

22. BV421 Streptoavidin (1:100, Biolegend, 405226)
The following antibodies were validated on manufacture's website:
https://www.biolegend.com/ja-jp/products/brilliant-violet-421-streptavidin-7297
We previously published use of this antibody in doi: 10.1016/j.stem.2017.10.011

Eukaryotic cell lines

Policy information about cell lines

Cell line source(s)	The ESC line (Hoxb7-GFP, clone #B6-5, male) was established in our previous study (Taguchi and Nishinakamura, 2017). G4-2 ESC line (male) expressing GFP ubiquitously (Niwa et al., 2000), which was kindly provided from Dr. Hitoshi Niwa at Kumamoto University.
Authentication	All mouse ES cell lines were authenticated by testing pluripotency gene expression.
Mycoplasma contamination	The ES cell lines are not tested for Mycoplasma contamination.
Commonly misidentified lines (See ICLAC register)	NO misidentified cell lines were used in this study.

Animals and other organisms

Policy information about studies involving animals; ARRIVE guidelines recommended for reporting animal research

Laboratory animals	Osr1GFP mice and Tbx18-MerCreMer mice were generated as described previously (Taguchi et al., 2014, Grisanti et al., 2013). Hoxb7-GFP mice (Yu et al., 2002), Foxd1-GFPCre mice (Kobayashi et al., 2014), Foxd1-GFPCreERT2 mice (Kobayashi et al., 2014), Isl1-MerCreMer mice (Laugwitz et al., 2005), and ROSA26-CAG-tdTomato mice (Madisen et al., 2010) were purchased from Jackson Laboratory. Foxd1-GFPCreERT2, Isl1-MerCreMer, and Tbx18-MerCreMer mice were maintained on C57BL/6 background, while the other strains were maintained on a mixed genetic background of C57BL/6 and ICR. Males of Osr1-GFP, Hoxb7-GFP, and Foxd1-GFPCre mice (2-8 months of age) were mated with female ICR mice (8-12 weeks of age) to obtain embryos. Males of Foxd1-GFPCreERT2, Isl1-MerCreMer, and Tbx18-MerCreMer mice (2-8 months of age) were mated with female ROSA26-CAG-tdTomato mice (8-12 weeks of age). For the transplantation experiments, immunodeficient 8–10-week-male mice (NOD/ShiJic-scidJcl) were purchased from Charles River Laboratory Japan, Inc. Mice were housed in a specific pathogen-free animal facility in plastic cages on a 12-h/12-h light/dark cycle, and fed an irradiated CE-2 diet (CLEA Japan Inc.). Mouse rooms were maintained at 22-23 degrees Celsius, with 40-60% humidity.
Wild animals	None of wild animals were used in the study

Field-collected samples	None of field-collected samples were used in the study
Ethics oversight	All animal experiments were performed in accordance with institutional ethical guidelines and approved by the licensing committee of Kumamoto University (approval numbers: A2019–113 and A2021-008).

Note that full information on the approval of the study protocol must also be provided in the manuscript.

Flow Cytometry

Plots

Confirm that:

- [x] The axis labels state the marker and fluorochrome used (e.g. CD4-FITC).
- [x] The axis scales are clearly visible. Include numbers along axes only for bottom left plot of group (a 'group' is an analysis of identical markers).
- [x] All plots are contour plots with outliers or pseudocolor plots.
- [x] A numerical value for number of cells or percentage (with statistics) is provided.

Methodology

Sample preparation	Induced organoids from embryonic tissues or mESCs were dissociated into single cells with 0.05% trypsin/EDTA and blocked with normal mouse serum. Cell surface marker staining was carried out in FACS buffer (1× HBSS containing 1% BSA and 0.035% $NaHCO_3$). Stained cells were washed and re-suspend by FACS buffer containing propidium iodide (PI). After filtrating the cell solution using a 70 µm strainer, the samples were ready for FACS analysis.
Instrument	FACS SORP Aria from BD biosciences
Software	The original FACS data sets were collected by using BD FACS Diva software (v8.0.1). Data analysis were performed with Flowjo software (ver 7.6.5, TreeStar).
Cell population abundance	The abundance of sorted fractions were indicated in Figures. Purity of the sorted cells were confirmed by qPCR analysis using specific markers.
Gating strategy	Live cells were initially gated with the FSC/SSC and PI-negative population, and then FSC-H/FSC-W was applied to exclude doublet cells. The isolated cell populations were subsequently gated with cell surface markers or GFP+ cells as indicated in the Figures.

- [x] Tick this box to confirm that a figure exemplifying the gating strategy is provided in the Supplementary Information.
